# Prospective associations between muscle strength and genetic susceptibility to type 2 diabetes with incident type 2 diabetes: a UK Biobank study

**DOI:** 10.1186/s12916-024-03819-9

**Published:** 2025-02-21

**Authors:** Mengyao Wang, Paul James Collings, Haeyoon Jang, Ziyuan Chen, Qiaoxin Shi, Hin Sheung Ho, Shan Luo, Shiu Lun Au Yeung, Youngwon Kim

**Affiliations:** 1 School of Public Health, The University of Hong Kong Li Ka Shing Faculty of Medicine, Room 301D 3/F, Jockey Club Building for Interdisciplinary Research, 5 Sassoon Road, Pokfulam, Hong Kong; 2 MRC Epidemiology Unit, University of Cambridge School of Clinical Medicine, Institute of Metabolic Science, Cambridge Biomedical Campus, Box 285, Cambridge, Cambridgeshire CB2 0QQ UK

**Keywords:** Muscle strength, Type 2 diabetes, Genetic susceptibility, UK Biobank

## Abstract

**Background:**

This study explored whether the prospective associations between muscle strength and incident type 2 diabetes (T2D) differ by varying levels of genetic susceptibility to T2D.

**Methods:**

This study included 141,848 white British individuals from the UK Biobank. Muscle strength was expressed as the relative value of grip strength (measured by a hand dynamometer) divided by fat-free mass (measured via bioelectrical impedance analysis). Three categories of muscle strength (low, medium and high) were generated based on the sex- and age-specific tertiles. Genetic risk of T2D was estimated using a weighted polygenic risk score based on 138 independent single-nucleotide polymorphisms for T2D. During a median 7.4-year follow-up, 4,743 incident T2D cases were accrued. Cox regression with age as the underlying timescale was fit.

**Results:**

High muscle strength was associated with a 44% lower hazard of T2D (HR:0.56, 95%CI:0.52–0.60), compared with low muscle strength, after adjustment for genetic risk of T2D. The inverse association between muscle strength and incident T2D was weaker in individuals with high genetic susceptibility. There was evidence of interaction between muscle strength and genetic susceptibility to T2D (p-additive = 0.010, p-multiplicative = 0.046). The estimated 8-year absolute risk of T2D was lower for high genetic risk—high muscle strength (2.47%), compared with low (2.89%) or medium (4.00%) genetic risk combined with low muscle strength.

**Conclusions:**

Higher muscle strength was associated with lower relative risk of developing T2D, irrespective of genetic susceptibility to T2D, while such association was weaker in the high genetic risk group. Individuals at high genetic risk of T2D but with high muscle strength may have a lower 8-year absolute risk of developing T2D, compared with those at low or medium genetic risk but with low muscle strength. Our findings inform future clinical trials to prevent or delay the onset of T2D by implementing muscle-strengthening interventions among individuals of varying levels of genetic susceptibility to T2D, including those with high genetic risk.

**Supplementary Information:**

The online version contains supplementary material available at 10.1186/s12916-024-03819-9.

## Background

Type 2 diabetes (T2D) is a complex, chronic metabolic disorder characterized by hyperglycemia due to insulin resistance and impaired insulin secretion [1–3]. T2D is associated with various complications [4, 5], such as cardiovascular disease, diabetic retinopathy, and diabetic neuropathy, leading to increased risks of disability and mortality [6]. Over the past few decades, the prevalence of T2D has been increasing substantially across the globe [7]. Estimates from the International Diabetes Federation in 2021 indicated that there were approximately 537 million people (accounting for over 10% of the global population) with diabetes worldwide, of whom 95% were people with T2D [7–9]. The prevalence is projected to increase to 783 million (accounting for over 12% of the global population) by 2045 [7, 9]. Preventing T2D has, therefore, become an urgent global public health task [10].

The etiology of T2D can be characterized as the interplay between non-modifiable genetic traits and modifiable lifestyle factors [3]. Regular physical activity, an essential healthy lifestyle behavior, can improve an individual’s physical fitness including muscular fitness [11, 12], thereby enhancing insulin sensitivity, improving glycemic traits, and, therefore, helping to prevent or delay incident T2D [13, 14]. Muscle strength, an important aspect of muscular fitness, has also been shown to be associated with lower risk of T2D [15]. Studies have indicated that the inverse association between muscle strength and incident T2D is independent of demographic, body composition and behavioral factors, such as age, adiposity, and lifestyle components [16–19]. However, to our knowledge, studies have not yet investigated whether the association between muscle strength and incident T2D is independent of, or might vary by, genetic susceptibility to T2D.

T2D is considered to be polygenic in nature where multiple genes of small effect contribute to individuals’ genetic susceptibility to T2D [20, 21]. The polygenic architecture of T2D has been disentangled through recent Genome-Wide Association Studies (GWAS) which have identified a series of single-nucleotide polymorphisms (SNPs) for T2D risk [20–22]. This information can be used to construct a polygenic risk score (PRS) to provide a quantitative metric of genetic predisposition to T2D [23, 24]. Two previous studies explored the interplay between physical activity and genetic susceptibility for T2D risk [25, 26]. While prior research investigated the combined associations of muscle strength and genetic risk with stroke outcomes [27], there is currently no evidence on the interplay of genetic susceptibility and muscle strength for metabolic disorders including T2D. Rigorous evidence is needed which could indicate whether improving muscle strength should be considered as a T2D prevention strategy in individuals with varying levels of genetic susceptibility to T2D particularly those with high genetic susceptibility to T2D. The purpose of this study was to investigate whether the associations between muscle strength and incident T2D may differ by genetic risk of T2D.

## Methods

### Study design and study population

We used data from UK Biobank which is an ongoing prospective cohort study of > 500,000 adults aged between 40 to 69 years. At baseline (conducted between 2006 to 2010), a wealth of information about socio-demographics, lifestyle behavior, as well as biological samples was collected through touch-screen questionnaires, nurse interview and physical measurements [28]. Participants have been followed for disease incidence and mortality via electronic linkage based on several data resources including UK primary care data, secondary care data (hospital admission records) and UK death registries since their first visit to the assessment center. In this analysis, we included 141,848 participants with valid genetic data, after excluding participants who: 1) did not self-report and genetically verified their ethnicity as white British, 2) had no primary care data, 3) had a prior history of diabetes (based on self-reported information of medical history and medication use) [29], 4) had a baseline glycated hemoglobin (HbA1c) concentration ≥ 48 mmol/mol or random glucose concentration ≥ 11.1 mmol/l [29], and 5) developed T2D in the first 2 years of follow-up; or 6) had missing information for any covariables. (see Additional file 1: Fig. S1) The protocol of UK Biobank was approved by the North West Multi-Centre Research Ethics Committee. The genetic substructure of the population was characterized by determining the first 20 principal components of genetic ancestry [30].

### Exposures

#### Muscle strength

In the UK Biobank, grip strength was included as an indicator of overall muscle strength [31–34]. Handgrip strength was measured using a hand dynamometer (Jamar hydraulic J00105) during the participants' first visit to the assessment center for baseline data collection. Sitting upright in a chair with the elbow at a 90-degree angle, participants were asked to squeeze the handle of the dynamometer as strongly as possible for approximately three seconds. Measurements were taken for both hands following the same protocol and the average value was calculated. Considering the potential impact of body size on grip strength, relative grip strength was derived as the quotient of absolute grip strength and body fat-free mass, measured via bioelectrical impedance [27, 35–37]. Following the established methodology [27, 38], we used age and sex-specific cut-off points for relative grip strength to categorize participants into three groups based on the tertiles (See Additional file 1: Table S1).

#### T2D genetic risk

Each participant’s genetic susceptibility to T2D was estimated by a PRS value. The PRS was calculated using PLINK 2.0 as the sum of the number of risk-increasing alleles at 138 genome-wide significant (*P* < 5 × 10^−8^) SNPs in low linkage disequilibrium with r^2^ < 0.001 (the list of alternative SNPs is provided in Additional file 1: Table S2), multiplied by the corresponding effect size obtained from the most recent GWAS for European ancestry [22]. The derived continuous PRS variable followed a normal distribution (Additional file 1: Fig. S2) and was categorized into three groups to signify low (bottom quintile), medium (middle three quintiles) and high (top quintile) T2D genetic risk [39].

### Study endpoint

We ascertained incident T2D cases via linkage of the UK Biobank data with UK primary care data, secondary care data (hospital admission records) and UK death registries. The primary care dataset is available for approximately 230,000 UK Biobank participants and contains data from general practitioner system suppliers [40]. Incidence of T2D was defined as the first occurrence of T2D cases accrued after baseline from the primary care, secondary care or mortality records. For primary care data, T2D events were determined based on the following 4 criteria: 1) a T2D diagnostic code (C10F); 2) diabetes medication use (insulin, metformin and non-metformin oral anti-diabetic agent); 3) hyperglycemia recorded on blood results (defined as HbA_1c_ ≥ 6.5% or 48 mmol/mol or fasting/random/unspecified glucose concentration ≥ 11.1 mmol/l); and 4) presence of ≥ 5 diabetes specific process of care codes (e.g., for foot screening) [29]. For secondary care and mortality data, T2D events were determined according to the Codes of International Classification of Disease [ICD 10: E11]. The date of T2D diagnosis was defined as the date of the first diagnosis of T2D as identified from either the primary care or secondary care data. Incident data were last censored on 31 March 2017 for Scotland, 31 May 2016 for England (TPP), 31 May 2017 for England (Vision) and 31 August 2017 for Wales [41]. Follow-up, which commenced from the participants’ first visit to the assessment center, was censored at these dates or at the time of T2D diagnosis. The median follow-up period was 7.4 years (interquartile range is 6.8–8.2 years). After excluding T2D cases that emerged in the first 2 years of follow-up, the final analytic sample included 4,743 incident T2D cases.

### Confounders

We included several demographic and lifestyle factors as potential confounders of associations: sex, the Townsend Deprivation Index (a continuous measure of area-based deprivation based on employment, car ownership, home ownership and household overcrowding), employment status (unemployed, paid employed/self-employed), tobacco smoking status (never, previous, current), alcohol drinking status (never, previous, currently less than 3 times/week, currently greater than or equal to 3 times/week), red meat intake (times/week; the average of weekly pork, beef and lamb/mutton intake), fish intake (< 2 times/week, ≥ 2 times/week) and physical activity (calculated based on self-reported walking, moderate physical activity and vigorous physical activity; MET minutes per week).

### Statistical analysis

To describe the sample, means and standard deviations for continuous variables and percentages for categorical variables were reported for all participants and stratified by categories of muscle strength. Multivariable Cox regression models with age as the underlying timescale were used to estimate hazard ratios (HRs) and 95% confidence intervals (CIs) for incident T2D. To explore the associations between muscle strength and incident T2D, we initially adjusted for all potential confounders (model 1) and then further adjusted T2D genetic risk, genotype array type and the first twenty principal components (model 2). Cox regression models specified with genetic risk of T2D as the exposure were adjusted for sex, genotype array type and the first twenty principal components. Adjusted for the same confounders, the cumulative hazards of T2D across the age range were plotted for each category of muscle strength and genetic risk of T2D, separately.

Multiplicative as well as additive interaction (Relative excess risk due to interaction [RERI] between covariates “A” [e.g. muscle strength] and “B” [T2D genetic risk] calculated as RR_A+B+_—RR_A+B—_RR_A-B+_ + 1) [42] between muscle strength and T2D genetic risk (both treated as ordinal variables) in relation to incident T2D were tested in the models that were adjusted for all confounders. To explore the association of muscle strength with incident T2D across different levels of T2D genetic risk, Cox regression models stratified by genetic risk of T2D were fit. The joint associations between muscle strength and genetic susceptibility to T2D with incident T2D were explored by generating a total of 9 combined categories of muscle strength and genetic risk of T2D, with the combined category of high muscle strength and low T2D genetic risk as the reference category in the Cox regression models. Estimated 8-year absolute risks of T2D were calculated for each category of muscle strength stratified by different levels of genetic risk of T2D using Cox regression models, adjusted for age, sex, genotype array type, and the first twenty principal components.

To accommodate familial clustering of data, second-degree or higher genetic relatedness (kinship coefficients between 0.0442 and 0.0884) was accounted for in all models by using cluster-robust standard errors. Log–log plots indicated that the proportional hazards assumption was met for each covariate. All statistical analyses were performed using Stata/SE Version 17 (StataCorp LP, College Station, TX) or R statistical software (RStudio 2022.02 + 485, Linux x86_64).

Eight sensitivity analyses were performed to evaluate the robustness of our findings. First, we excluded T2D cases that were developed within an additional 2 years of follow-up (4 years in total) to further reduce the possibility of reverse causality. Second, we retained in the analysis only participants who did not exhibit second-degree or higher genetic relatedness and kept at random only one participant from each familial cluster. Third, we replicated the analysis with absolute grip strength as an exposure. Fourth, we used a less stringent clumping* r*^*2*^ threshold in the SNP selection procedure (i.e., linkage disequilibrium at *r*^*2*^ < 0.01, the list of alternative SNPs is provided in Additional file 1: Table S3; the derived continuous PRS variable followed a normal distribution [Additional file 1: Fig. S3]). Fifth, we ascertained incident T2D based only on primary care data and defined the incident date of T2D as the mid-point between the last primary care consultation without T2D and the date of the first T2D [29]. Sixth, we ascertained incident T2D based only on hospital admission records and UK death registries data. Seventh, we used a multiple imputation method to deal with the missing covariates (assuming data missing at random; Additional file 1: Table S4). Eighth, we categorized muscle strength in another way by classifying it into 5 categories based on the quintiles [17].

## Results

Among all 141,848 participants included in the final analysis, 54.8% of them were female and the average age was 56.6 years. Detailed participant characteristics for the full sample and for each category of muscle strength and genetic risk of T2D are presented in Table 1.

**Table 1:** Participants’ baseline information

Variables	All (*n* = 141,848)	Categories of muscle strength			Genetic risk of type 2 diabetes		
		Low (*n* = 47,221)	Medium (*n* = 47,286)	High (*n* = 47,341)	Low (*n* = 28,367)	Medium (*n* = 85,106)	High (*n* = 28,375)
Age, years	56.6 (8.0)	56.8 (8.0)	56.6 (8.0)	56.3 (8.0)	56.7 (8.0)	56.6 (8.0)	56.5 (8.0)
Sex, %							
Women	77,751 (54.8)	25,889 (54.8)	25,912 (54.8)	25,950 (54.8)	15,386 (54.2)	46,754 (54.9)	15,611 (55.0)
Men	64,097 (45.2)	21,332 (45.2)	21,374 (45.2)	21,391 (45.2)	12,981 (45.8)	38,352 (45.1)	12,764 (45.0)
Body mass index, kg/m^2^	27.2 (4.5)	28.8 (5.2)	27.1 (4.2)	25.8 (3.6)	27.3 (4.6)	27.3 (4.5)	27.2 (4.4)
Townsend Deprivation Index	−1.6 (2.9)	−1.2 (3.0)	−1.7 (2.8)	−1.9 (2.7)	−1.6 (2.9)	−1.6 (2.9)	−1.6 (2.9)
Employment status, %							
Not in paid employment	59,874 (42.2)	21,417 (45.4)	19,492 (41.2)	18,965 (40.1)	12,165 (42.9)	35,865 (42.1)	11,844 (41.7)
Paid employed/self-employed	81,974 (57.8)	25,804 (54.6)	27,794 (58.8)	28,376 (59.9)	16,202 (57.1)	49,241 (57.9)	16,531 (58.3)
Smoking status, %							
Never	78,827 (55.7)	25,857 (54.8)	26,275 (55.6)	26,695 (56.4)	15,805 (55.7)	46,994 (55.2)	16,028 (56.5)
Previous	48,693 (34.3)	16,485 (34.9)	16,422 (34.7)	15,786 (33.3)	9,743 (34.4)	29,459 (34.6)	9,491 (33.5)
Current	14,328 (10.0)	4,879 (10.3)	4,589 (9.7)	4,860 (10.3)	2819 (9.9)	8,653 (10.3)	2,856 (10.0)
Alcohol consumption, %							
Never	4,344 (3.0)	1,709 (3.6)	1,372 (2.9)	1,263 (2.7)	899 (3.2)	2,609 (3.1)	836 (3.0)
Previous	4,667 (3.2)	1,958 (4.2)	1,408 (3.0)	1,191 (2.5)	904 (3.2)	2,772 (3.2)	881 (3.1)
Current (< 3times/week)	68,736 (48.5)	24,330 (51.5)	22,713 (48.0)	21,693 (45.8)	13,619 (48.0)	41,148 (48.4)	13,970 (49.2)
Current (≥ 3times/week)	68,211 (45.3)	19,224 (40.7)	21,793 (46.1)	23,194 (49.0)	12,946 (45.6)	38,577 (45.3)	12,688 (44.7)
Average red meat intake, times/week	0.44 (0.35)	0.44 (0.37)	0.44 (0.35)	0.43 (0.34)	0.44 (0.35)	0.44 (0.35)	0.44 (0.35)
Fish intake ≥ 2 times/week, %	72,659 (51.2)	23,445 (49.7)	24,451 (51.7)	24,763 (52.3)	14,503 (51.1)	43,631 (51.3)	14,525 (51.2)
Physical activity (MET-minutes/week)	372.9 (375.4)	344.5 (364.8)	375.6 (374.5)	398.4 (384.2)	372.4 (377.8)	372.2 (374.8)	375.3 (375.0)

Values are means (standard deviations) or percentages unless otherwise indicated

### Independent associations of muscle strength and genetic risk of T2D with T2D

**Fig. 1:**
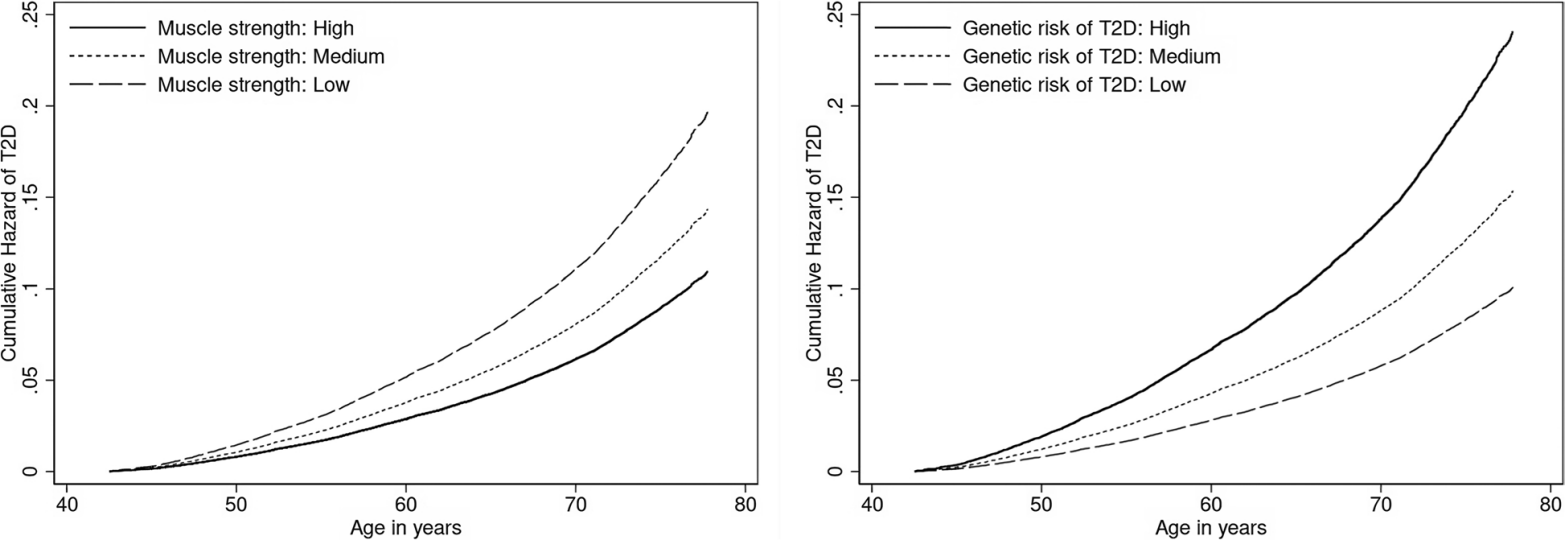
Cumulative hazard rates of type 2 diabetes according to categories of muscle strength and categories of genetic risk of type 2 diabetes. Note: Cox regression model with categories of muscle strength as the exposure and incorporating age as the underlying time scale were adjusted for sex, Townsend Deprivation Index, employment status, tobacco smoking status, alcohol drinking status, red meat intake, fish intake, physical activity, polygenic risk score for T2D, genotype array type and the first twenty principal components. Cox regression model with categories of genetic risk of T2D as the exposure and incorporating age as the underlying time scale were adjusted for sex, genotype array type and the first twenty principal components. Abbreviations: T2D, type 2 diabetes

Table 2 shows the independent associations of muscle strength and genetic susceptibility to T2D with incident T2D. Compared with the lowest tertile of muscle strength, the medium and highest tertiles were associated with 27% (HR: 0.73, 95%CI: 0.68–0.78) and 44% (HR:0.56, 95%CI: 0.52–0.60) lower risk of T2D, respectively, independent of potential confounders (Model 1). The inverse association between muscle strength and incident T2D remained strong after additional adjustment for PRS for T2D, genotype array type and the first twenty principal components. Greater genetic risk of T2D was associated with a higher risk of incident T2D. The HR of incident T2D was 1.52 (95%CI: 1.40–1.66) and 2.39 (95%CI: 2.17–2.63) for medium and high genetic risk categories, respectively, compared with the low T2D genetic risk category. The cumulative hazards of T2D according to the muscle strength and genetic risk of T2D across the age range were presented in Fig. 1. Considerably higher cumulative hazards of T2D were observed across descending categories of muscle strength and ascending categories of genetic risk of T2D.

**Table 2:** Associations of muscle strength and genetic susceptibility to type 2 diabetes with incident type 2 diabetes

Comparison	Number of participants	Number of cases	Crude incident rate per 100,000-person years	Hazard ratio (95% confidence interval)	
				Model 1	Model 2
	141,848	4,743	442.7		
Categories of muscle strength					
Low (Reference)	47,221	2,119	606.8	1 (Reference)	1 (Reference)
Medium	47,286	1,487	417.8	0.73 (0.68, 0.78)	0.73 (0.68, 0.78)
High	47,341	1,137	310.3	0.56 (0.52, 0.60)	0.56 (0.52, 0.60)
Categories of genetic risk of T2D					
Low (Reference)	28,367	690	283.1	1 (Reference)	
Medium	85,106	2,733	424.8	1.52 (1.40, 1.66)	-
High	28,375	1,401	657.6	2.39 (2.17, 2.63)	-

Model 1 using categories of muscle strength as the exposure and incorporating age as the underlying time scale were adjusted for sex, Townsend Deprivation Index, employment status, tobacco smoking status, alcohol drinking status, red meat intake, fish intake and physical activity. Model 2 using categories of muscle strength as the exposure was adjusted for all confounders in Model 1 and additionally adjusted for polygenic risk score for T2D, genotype array type and the first twenty principal components. Model 1 using categories of genetic risk of T2D as the exposure and incorporating age as the underlying time scale were adjusted for genotype array type and the first twenty principal components. Abbreviations: T2D, type 2 diabetes

### Joint associations of muscle strength and genetic risk of T2D with T2D

**Fig. 2:**
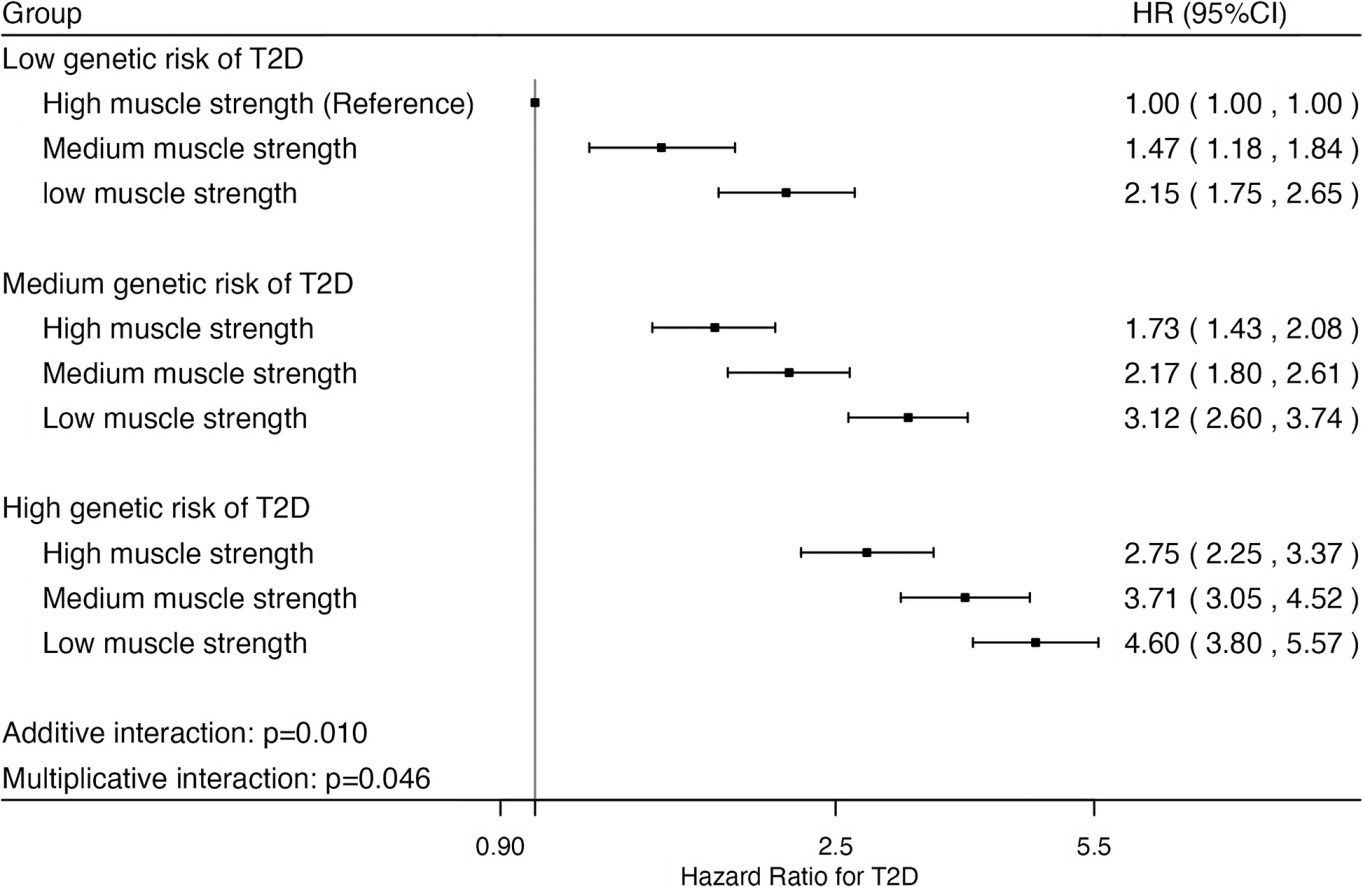
Joint association between categories of muscle strength and genetic risk of type 2 diabetes with incident type 2 diabetes. Note: Models with the age as the underlying timescale were adjusted for sex, Townsend Deprivation Index, employment status, tobacco using status, alcohol drinking status, red meat intake, fish intake, physical activity, genotype array type and the first twenty principal components. The p-value for additive and multiplicative interaction between muscle strength and genetic risk of T2D is 0.010 and 0.046, respectively. Abbreviation: T2D, type 2 diabetes; HR, hazard ratio; CI, confidence interval

Table 3 shows the associations between muscle strength and incident T2D stratified by different levels of T2D genetic risk. Within each level of T2D genetic risk, the highest tertile of muscle strength was consistently associated with lower hazard of T2D, compared with the lowest tertile of muscle strength. However, we found that the inverse association between muscle strength and incident T2D was relatively weaker at high T2D genetic risk than at low T2D genetic risk. Figure 2 presents the joint association between muscle strength and genetic risk of T2D with incident T2D. Within 9 joint categories of muscle strength and genetic risk of T2D, the combined category of high T2D genetic risk and low muscle strength was associated with the highest hazards of T2D (HR: 4.60, 95%CI: 3.80–5.57), compared with the combined category of low T2D genetic risk and high muscle strength. Notably, evidence of interaction between muscle strength and genetic susceptibility to T2D was observed on both additive (*p* = 0.010) and multiplicative (*p* = 0.046) scales where the associations between muscle strength and incident T2D were relatively stronger at low genetic risk than at high genetic risk. The results of the sensitivity analyses were, in general, similar to the results of the main analysis (Additional file 1: Table S5-S12).

**Table 3:** Associations of muscle strength and incident type 2 diabetes stratified by different levels of genetic susceptibility to type 2 diabetes

Categories of T2D genetic risk	Categories of muscle strength	Number of Participants	Number of T2D events	Crude incident rate per 100,000-person years	HR (95% CI)	P for interaction^1^
Low	Low (Reference)	9,380	288	412.5	1 (Reference)	Additive: 0.010 Multiplicative: 0.046
	Medium	9,513	193	268.4	0.69 (0.57, 0.82)	
	High	9,474	128	174.4	0.47 (0.38, 0.58)	
Medium	Low (Reference)	28,385	1240	590.6	1 (Reference)	
	Medium	28,406	835	390.5	0.71 (0.65, 0.77)	
	High	28,315	658	299.7	0.56 (0.51, 0.62)	
High	Low (Reference)	9,456	591	851.2	1 (Reference)	
	Medium	9,367	459	654.3	0.80 (0.71, 0.91)	
	High	9,552	351	477.8	0.59 (0.52, 0.68)	

Cox regression models with age as the underlying timescale were adjusted for sex, Townsend Deprivation Index, employment status, tobacco smoking status, alcohol drinking status, red meat intake, fish intake, physical activity, genotype array type and the first twenty principal components. Abbreviations: T2D, type 2 diabetes; HR, hazard ratio; CI, confidence interval

^1^P for interaction: the *p*-value for the interaction term between categories of muscle strength and genetic risk for T2D (both treated as ordinal variables) for incident T2D on additive and multiplicative scales

### 8-year absolute risk of T2D

**Fig. 3:**
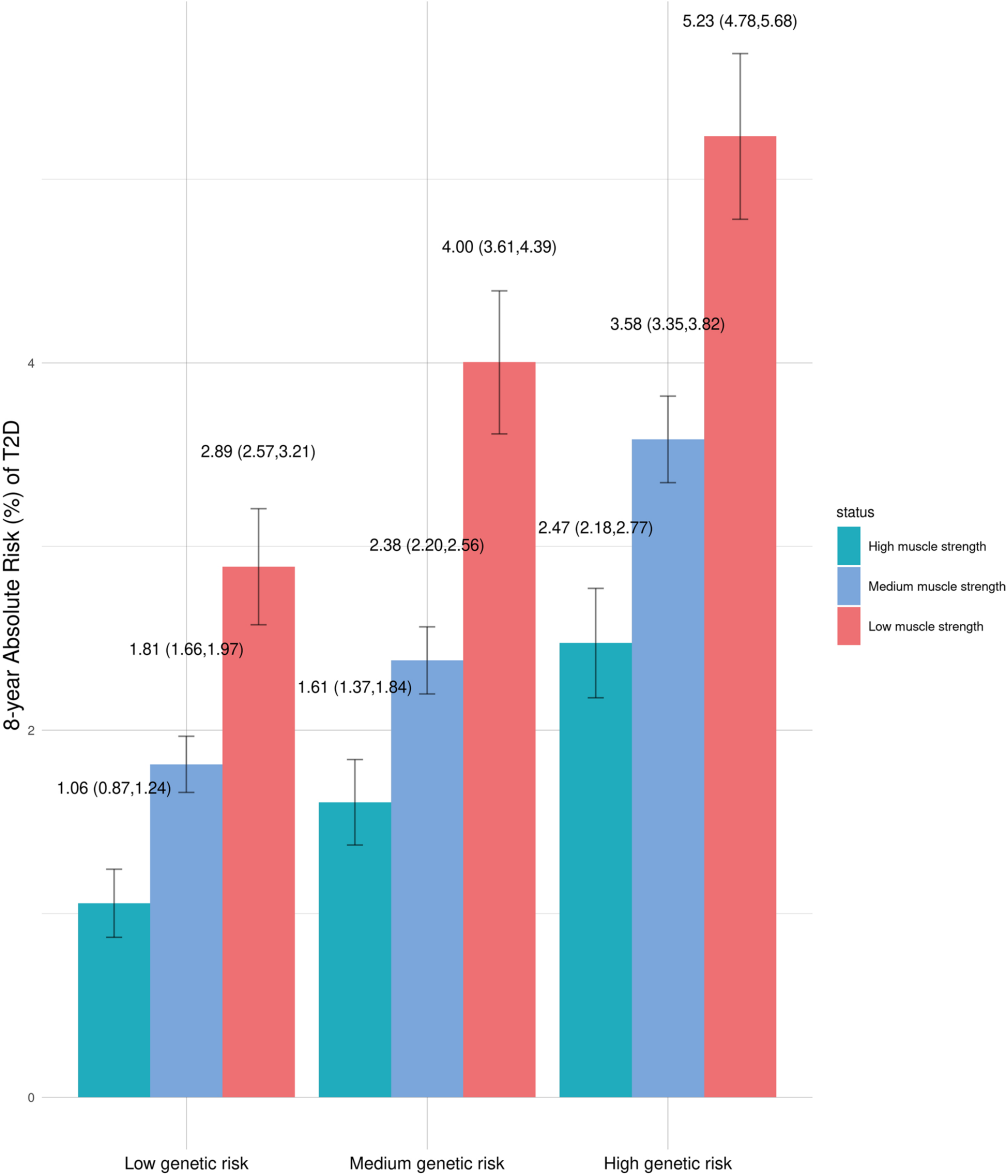
Estimated 8-year absolute risk of incident type 2 diabetes stratified by categories of muscle strength and genetic risk of type 2 diabetes. Note: Cox regression models were adjusted for age, sex, genotype array type, and the first twenty principal components. Error bars: 95% confidence interval for the estimated 8-year absolute risk. Abbreviations: T2D, type 2 diabetes

Figure 3 shows the estimated 8-year absolute risk of T2D for each category of muscle strength across different levels of T2D genetic risk. The 8-year absolute risk of T2D ranged from 1.06% to 2.89% for low T2D genetic risk, from 1.61% to 4.00% for medium genetic risk, and 2.47% to 5.23% for high genetic risk. Notably, high T2D genetic risk combined with high muscle strength had lower 8-year absolute risk of T2D (2.47%) than medium (2.89%) or low (4.00%) genetic risk combined with low muscle strength.

## Discussion

To our knowledge, this is the first study to explore the prospective associations between muscle strength and genetic susceptibility to T2D with respect to the risk of T2D. We found that higher genetic risk of T2D was strongly linked to the future risk of developing T2D. Muscle strength was inversely associated with the risk of T2D, while such associations were weaker in the high T2D genetic risk group. The estimated 8-year absolute of T2D was lower for individuals at high genetic risk of T2D but with high muscle strength compared to those at low genetic risk of T2D but with low muscle strength.

The inverse association between muscle strength and incident T2D corroborates findings of several previous prospective studies and meta-analyses [16–19]. Our findings indicated that the highest tertile of muscle strength could have nearly 50% lower hazards of incident T2D compared to the lowest tertile even after taking into account genetic susceptibility to T2D as well as potential confounders. The underlying mechanism for the inverse association between muscle strength and incident T2D has not been fully elucidated. However, evidence indicates that loss of muscle mass and muscle strength can potentially lead to the decreased surface area of glucose transport and the potential worsening of insulin resistance [43]. Meanwhile, as one of the major determinants of muscle strength, participation in muscle-strengthening activities has been shown to improve insulin sensitivity and increase glucose transport through decreasing skeletal muscle insulin resistance by increasing skeletal muscle mitochondria and glucose transporter type 4 (GLUT4) protein expression [44, 45]. Findings from Mendelian randomization research also suggested that muscle mass and muscle strength may have causal associations with diabetes [16]. Taken together, these findings suggest that maintaining or improving muscle strength could be considered an important strategy in T2D prevention.

Moreover, the p-values for interaction terms hinted at the potential role of muscle strength as an effect modifier in the associations between genetic risk and incident T2D. Of note, the relative risk (i.e. hazard ratio) of T2D for muscle strength was, in general, greater in individuals at low genetic risk of T2D than in those at higher genetic risk, with p-values for interaction indicative of potential interaction; p-value: 0.010 for additive interaction and 0.046 for multiplicative interaction. The underlying mechanisms and causal evidence of the interaction remain uncertain. The high genetic risk of T2D contributes to an overall high risk of developing the condition, which may have led to the less pronounced associations of high muscle strength with T2D risk for these individuals at high genetic risk of T2D. To the best of our knowledge, no previous research has explored such interactions between muscle strength, genetic risk, and T2D risk, therefore no direct comparison can be made possible. However, two studies have reported evidence of interaction between physical activity and genetic susceptibility to T2D with incident T2D, indicating that the protective effects of physical activity against developing T2D were weaker in individuals with a high genetic risk [25, 26]. Future clinical trials are warranted to determine the extent to which resistance exercise or muscle-strengthening activities elicit favorable impacts on T2D risk and metabolic risk markers in individuals of different levels of genetic risk of T2D.

It is also notable that individuals at high genetic risk of T2D but with high muscle strength displayed a lower 8-year absolute risk of T2D, compared to individuals at low or medium genetic risk but with low muscle strength. This finding suggests that while the relative risk of T2D may be weaker at high genetic risk, it would be important, from a public health perspective, to target individuals at high genetic risk of T2D who, compared with those at low genetic risk, may have a substantially higher absolute risk of T2D, but may receive a greater benefit in their absolute risk of developing T2D through participation in muscle-strengthening activities [46, 47]. Therefore, from a public health perspective, targeting individuals at high genetic risk of T2D through the utilization of PRS for T2D and then maintaining or improving their muscle strength may be a feasible strategy for T2D prevention [23, 24]. Previous clinical trials have demonstrated the favorable impacts of resistance exercise or training on markers of T2D in average adults [44, 45], but future experimental research is warranted to provide rigorous evidence on the degree to which regular participation in muscle-strengthening activities could reduce the predicted estimate of absolute risk of developing T2D among individuals of varying levels of genetic risk for T2D, as identified by PRS for T2D.

There are several strengths and limitations of our study worth noting. We used large-scale prospective cohort data with a relatively long follow-up period to explore the interplay between muscle strength and genetic susceptibility to T2D for incident T2D. To achieve more rigorous adjudication of incident T2D, we utilized a combination of multiple data sources including primary care data, hospital admission records, and the UK death registries. However, we used the diagnosis date of T2D in our analyses since it was not possible to determine the exact date of T2D onset due to the observational nature of the UK Biobank project [29]. In this sense, while comparisons of absolute risk across different categories may be fair, the estimated 8-year absolute risk of T2D may be subject to potential bias. Meanwhile, as the reported associations are based on observational data, causal evidence cannot be inferred in this study. Moreover, although several confounders were adjusted for, there may still exist residual confounding due to measurement error or unmeasured confounders. Our study only included participants of European ancestry. Therefore, it is important to note that the generalizability of our findings to other populations may be limited. Moreover, the potential selection bias in the UK Biobank should be acknowledged [48].

## Conclusions

Higher muscle strength was associated with lower relative risk of developing T2D, irrespective of genetic risk of T2D. Evidence of potential additive (p-value: 0.010) and multiplicative (p-value: 0.046) interactions between muscle strength and genetic risk of T2D suggests that muscle strength has the potential to act as an effect modifier in the pathways of T2D genetic risk towards incident T2D. Nonetheless, individuals at high genetic risk of T2D but with high muscle strength may have a lower 8-year absolute risk of T2D compared with those at low or medium genetic risk but with low muscle strength. Therefore, it would be advantageous from a public health perspective to improve or maintain muscle strength for T2D prevention targeting genetically at-risk individuals. Our findings inform future clinical trials and policies to prevent or delay the onset of T2D by implementing muscle-strengthening intervention among individuals of varying levels of genetic susceptibility to T2D, especially those at high genetic risk.

## Supplementary Information

Additional file 1: Table S1-S10 and Figure S1-S3. Table S1. Age- and sex-specific cut-points used to create tertiles of muscle strength. Table S2-S3. Lists of Single-Nucleotide Polymorphisms related to type 2 diabetes. Table S4. Multiple imputation for missing data. Table S5-11. Results from sensitivity analyses. Fig S1. Participants’ flow chart. Fig S2-3. Distribution of polygenic risk scores for type 2 diabetes.

## Data Availability

The data that support the findings of this study are available from the UK Biobank but restrictions apply to the availability of these data, which were used under license for the current study, and so are not publicly available. Data are however available from the authors upon reasonable request and with permission of the UK Biobank.

## Abbreviations

T2D: Type 2 diabetes
GWAS: Genome-Wide Association Studies
SNPs: Single-nucleotide polymorphisms
PRS: Polygenic risk scores
HbA1c: Glycated hemoglobin
HR: Hazard ratio
CI: Confidence interval
