# Supplementary material for: Prospective associations between muscle strength and genetic susceptibility to type 2 diabetes with incident type 2 diabetes: a UK Biobank study
Source: BMC Med. 2025 Feb 21;23:93. doi: 10.1186/s12916-024-03819-9 (PMC11846218; doi:10.1186/s12916-024-03819-9)
Supplement: Supplementary file 1 — Additional file 1: Table S1-S10 and Figure S1-S3. Table S1. Age- and sex-specific cut-points used to create tertiles of muscle strength. Table S2-S3. Lists of Single-Nucleotide Polymorphisms related to type 2 diabetes. Table S4. Multiple imputation for missing data. Table S5-11. Results from sensitivity analyses. Fig S1. Participants’ flow chart. Fig S2-3. Distribution of polygenic risk scores for type 2 diabetes. [file 12916_2024_3819_MOESM1_ESM.docx]

# Additional file 1

**Table S1.** Age- and sex-specific cut-points used to create tertiles of muscle strength.

**Table S2-3.** Lists of Single-Nucleotide Polymorphisms (SNPs) related to type 2 diabetes.

**Table S4.** Multiple imputation for missing data.

**Table S5-12.** Results from sensitivity analyses.

**Figure S1.** Participants’ flow chart

**Figure S2-3.** Distribution of polygenic risk scores for type 2 diabetes.

# Table S1. Age- and sex-specific cut-points used to create tertiles of muscle strength.

| Age range | Tertile of muscle strength | Men* | Women* |
| --- | --- | --- | --- |
| <50 years | Low | < 0.61 | <0.54 |
|  | Middle | 0.61-0.71 | 0.54-0.64 |
|  | High | ≥0.72 | ≥0.65 |
| 50-59 years | Low | <0.59 | < 0.48 |
|  | Middle | 0.59-0.68 | 0.48-0.58 |
|  | High | ≥0.69 | ≥0.59 |
| ≥60 years | Low | <0.56 | <0.45 |
|  | Middle | 0.56-0.65 | 0.45-0.54 |
|  | High | ≥0.66 | ≥0.55 |

*Grip strength (kg) divided by fat-free mass (kg)

Table S2. A list of 138 Single-Nucleotide Polymorphism (SNPs) known to be associated with type 2 diabetes genome-wide significant at a *p*-value of 5×10^-8^ and in low linkage disequilibrium defined according to *r^2^*<0.001.

| SNP | Effect allele | Other allele | Beta | P-value |
| --- | --- | --- | --- | --- |
| rs10811661 | T | C | 0.177 | 1.90E-132 |
| rs76895963 | T | G | 0.532 | 8.30E-86 |
| rs9348441 | A | T | 0.137 | 5.50E-83 |
| rs55872725 | T | C | 0.122 | 8.50E-79 |
| rs7633675 | G | T | 0.109 | 3.20E-57 |
| rs13266634 | C | T | 0.108 | 1.40E-54 |
| rs10830963 | G | C | 0.104 | 3.00E-48 |
| rs10226758 | C | A | 0.069 | 1.50E-44 |
| rs2943648 | G | A | 0.091 | 3.30E-43 |
| rs231361 | A | G | 0.093 | 5.90E-40 |
| rs9998835 | G | C | 0.084 | 3.80E-37 |
| rs17036160 | C | T | 0.115 | 1.00E-33 |
| rs256904 | T | A | 0.085 | 5.20E-33 |
| rs10908278 | T | A | 0.079 | 5.20E-32 |
| rs952472 | C | A | 0.081 | 5.50E-31 |
| rs77464186 | A | C | 0.1 | 9.30E-31 |
| rs11708067 | A | G | 0.088 | 5.00E-30 |
| rs115505614 | T | C | 0.171 | 4.70E-29 |
| rs11257655 | T | C | 0.086 | 1.50E-28 |
| rs13414140 | C | T | 0.113 | 5.10E-27 |
| rs703980 | G | A | 0.07 | 8.10E-27 |
| rs5215 | C | T | 0.071 | 9.80E-27 |
| rs72802358 | G | C | 0.114 | 2.20E-26 |
| rs9379084 | G | A | 0.11 | 4.30E-26 |
| rs2796441 | G | A | 0.068 | 8.00E-26 |
| rs3768301 | T | C | 0.079 | 2.80E-25 |
| rs61953351 | G | T | 0.072 | 6.40E-24 |
| rs2215383 | C | T | 0.064 | 7.60E-24 |
| rs28429551 | A | T | 0.078 | 2.60E-23 |
| rs3887925 | T | C | 0.059 | 2.90E-23 |
| rs1260326 | C | T | 0.064 | 1.60E-22 |
| rs3806155 | T | A | 0.176 | 3.30E-22 |
| rs1800961 | T | C | 0.166 | 2.70E-21 |
| rs8192675 | T | C | 0.066 | 5.80E-21 |
| rs12578595 | C | T | 0.074 | 3.10E-20 |
| rs243018 | G | C | 0.059 | 1.80E-19 |
| rs10184004 | C | T | 0.059 | 2.00E-19 |
| rs6496609 | C | A | 0.064 | 2.80E-19 |
| rs34715063 | C | T | 0.088 | 5.00E-19 |
| rs340874 | C | T | 0.055 | 1.10E-18 |
| rs2366214 | A | G | 0.059 | 1.70E-18 |
| rs7732130 | G | A | 0.061 | 5.70E-18 |
| rs11759026 | G | A | 0.063 | 4.50E-17 |
| rs343093 | G | C | 0.075 | 1.60E-16 |
| rs17168486 | T | C | 0.068 | 3.30E-16 |
| rs11688682 | G | C | 0.061 | 5.80E-16 |
| rs6813195 | C | T | 0.058 | 7.00E-16 |
| rs1562396 | G | A | 0.056 | 9.60E-16 |
| rs6567160 | C | T | 0.061 | 1.00E-15 |
| rs35895680 | C | A | 0.055 | 1.10E-15 |
| rs13094957 | T | C | 0.063 | 1.60E-15 |
| rs878521 | A | G | 0.059 | 2.30E-15 |
| rs429358 | T | C | 0.072 | 2.50E-15 |
| rs10406431 | A | G | 0.05 | 3.80E-15 |
| rs835576 | C | T | 0.079 | 5.80E-15 |
| rs7081841 | G | C | 0.053 | 6.00E-15 |
| rs2925979 | T | C | 0.055 | 7.10E-15 |
| rs13257021 | A | G | 0.049 | 3.10E-14 |
| rs3798519 | C | A | 0.062 | 4.60E-14 |
| rs1996546 | G | T | 0.069 | 8.90E-14 |
| rs12789028 | A | G | 0.061 | 1.10E-13 |
| rs6458354 | C | T | 0.052 | 1.40E-13 |
| rs348330 | G | A | 0.049 | 2.10E-13 |
| rs13290396 | C | T | 0.094 | 2.30E-13 |
| rs3890400 | A | G | 0.05 | 3.00E-13 |
| rs508419 | G | A | 0.05 | 3.10E-13 |
| rs8008910 | A | G | 0.055 | 4.20E-13 |
| rs58542926 | T | C | 0.087 | 4.70E-13 |
| rs2292662 | C | T | 0.064 | 4.80E-13 |
| rs2890156 | A | T | 0.066 | 6.00E-13 |
| rs7163757 | C | T | 0.047 | 7.90E-13 |
| rs66815886 | G | T | 0.051 | 8.70E-13 |
| rs7313668 | T | G | 0.046 | 9.90E-13 |
| rs6485462 | C | T | 0.049 | 1.60E-12 |
| rs1426371 | G | A | 0.052 | 1.70E-12 |
| rs2115107 | A | G | 0.047 | 1.70E-12 |
| rs6752053 | T | C | 0.045 | 3.20E-12 |
| rs36575 | C | T | 0.08 | 3.30E-12 |
| rs6937795 | A | C | 0.045 | 3.70E-12 |
| rs505922 | C | T | 0.047 | 3.70E-12 |
| rs6600191 | T | C | 0.059 | 4.50E-12 |
| rs8071043 | C | T | 0.047 | 7.50E-12 |
| rs11819995 | T | C | 0.053 | 1.10E-11 |
| rs12811407 | A | G | 0.048 | 1.20E-11 |
| rs539515 | C | A | 0.055 | 1.40E-11 |
| rs6091115 | T | C | 0.044 | 1.90E-11 |
| rs41277236 | T | C | 0.112 | 2.00E-11 |
| rs1426385 | A | G | 0.043 | 2.30E-11 |
| rs4240673 | T | C | 0.043 | 2.30E-11 |
| rs3111316 | A | G | 0.044 | 3.50E-11 |
| rs62107261 | T | C | 0.105 | 6.50E-11 |
| rs78470967 | T | A | 0.113 | 6.60E-11 |
| rs1108646 | A | G | 0.044 | 6.80E-11 |
| rs879882 | C | T | 0.042 | 7.20E-11 |
| rs13130484 | T | C | 0.044 | 8.50E-11 |
| rs145678014 | G | T | 0.105 | 1.40E-10 |
| rs736266 | T | A | 0.041 | 1.40E-10 |
| rs3094515 | C | T | 0.048 | 1.60E-10 |
| rs4911405 | T | C | 0.043 | 2.90E-10 |
| rs11642430 | G | C | 0.041 | 3.30E-10 |
| rs73221123 | T | C | 0.098 | 4.00E-10 |
| rs9899520 | A | G | 0.05 | 4.20E-10 |
| rs539298 | A | G | 0.04 | 4.60E-10 |
| rs2858980 | G | A | 0.054 | 4.60E-10 |
| rs4237150 | C | G | 0.04 | 4.80E-10 |
| rs6821438 | A | G | 0.04 | 5.10E-10 |
| rs10842708 | G | A | 0.045 | 6.40E-10 |
| rs6884702 | G | A | 0.04 | 7.90E-10 |
| rs10893827 | A | G | 0.056 | 9.00E-10 |
| rs917195 | C | T | 0.047 | 1.00E-09 |
| rs12001437 | C | T | 0.04 | 1.10E-09 |
| rs7178762 | C | T | 0.039 | 1.40E-09 |
| rs7781557 | C | T | 0.05 | 1.60E-09 |
| rs30614 | A | G | 0.039 | 1.70E-09 |
| rs12380322 | G | A | 0.039 | 1.90E-09 |
| rs57676627 | T | C | 0.057 | 2.00E-09 |
| rs10406327 | C | G | 0.038 | 2.40E-09 |
| rs28691713 | C | T | 0.041 | 2.40E-09 |
| rs12345069 | C | T | 0.043 | 3.40E-09 |
| rs12073283 | C | G | 0.066 | 3.60E-09 |
| rs4709746 | C | T | 0.057 | 4.00E-09 |
| rs73347525 | A | G | 0.05 | 4.90E-09 |
| rs1665901 | A | T | 0.04 | 5.00E-09 |
| rs9873519 | T | C | 0.037 | 6.30E-09 |
| rs10471048 | G | C | 0.039 | 6.40E-09 |
| rs34965774 | A | G | 0.054 | 6.40E-09 |
| rs1412234 | C | T | 0.039 | 7.70E-09 |
| rs4733612 | G | A | 0.042 | 9.20E-09 |
| rs12920022 | A | T | 0.053 | 1.00E-08 |
| rs7594480 | T | C | 0.075 | 1.10E-08 |
| rs329122 | A | G | 0.037 | 1.70E-08 |
| rs445084 | G | A | 0.038 | 2.00E-08 |
| rs2624847 | G | T | 0.041 | 3.50E-08 |
| rs963740 | A | T | 0.039 | 3.70E-08 |
| rs223423 | G | A | 0.035 | 4.30E-08 |
| rs10882099 | T | C | 0.019 | 4.50E-08 |
| rs12883788 | T | C | 0.035 | 4.60E-08 |
| rs7903146 | T | C | 0.298 | 1.00E-200 |

Table S3. A list of 187 Single-Nucleotide Polymorphism (SNPs) known to be associated with type 2 diabetes genome-wide significant at a *p*-value of 5×10^-8^ and in low linkage disequilibrium defined according to *r^2^*<0.01.

| SNP | Effect allele | Other allele | Beta | P-value |
| --- | --- | --- | --- | --- |
| rs10811661 | T | C | 0.177 | 1.90E-132 |
| rs76895963 | T | G | 0.532 | 8.30E-86 |
| rs9348441 | A | T | 0.137 | 5.50E-83 |
| rs55872725 | T | C | 0.122 | 8.50E-79 |
| rs7633675 | G | T | 0.109 | 3.20E-57 |
| rs13266634 | C | T | 0.108 | 1.40E-54 |
| rs10830963 | G | C | 0.104 | 3.00E-48 |
| rs10226758 | C | A | 0.069 | 1.50E-44 |
| rs2943648 | G | A | 0.091 | 3.30E-43 |
| rs231361 | A | G | 0.093 | 5.90E-40 |
| rs9998835 | G | C | 0.084 | 3.80E-37 |
| rs3217792 | C | T | 0.145 | 1.70E-34 |
| rs17036160 | C | T | 0.115 | 1.00E-33 |
| rs256904 | T | A | 0.085 | 5.20E-33 |
| rs10908278 | T | A | 0.079 | 5.20E-32 |
| rs952472 | C | A | 0.081 | 5.50E-31 |
| rs77464186 | A | C | 0.1 | 9.30E-31 |
| rs11708067 | A | G | 0.088 | 5.00E-30 |
| rs115505614 | T | C | 0.171 | 4.70E-29 |
| rs11257655 | T | C | 0.086 | 1.50E-28 |
| rs72830009 | G | A | 0.218 | 5.10E-28 |
| rs13414140 | C | T | 0.113 | 5.10E-27 |
| rs703980 | G | A | 0.07 | 8.10E-27 |
| rs5215 | C | T | 0.071 | 9.80E-27 |
| rs72802358 | G | C | 0.114 | 2.20E-26 |
| rs9379084 | G | A | 0.11 | 4.30E-26 |
| rs2796441 | G | A | 0.068 | 8.00E-26 |
| rs3768301 | T | C | 0.079 | 2.80E-25 |
| rs61953351 | G | T | 0.072 | 6.40E-24 |
| rs2215383 | C | T | 0.064 | 7.60E-24 |
| rs28429551 | A | T | 0.078 | 2.60E-23 |
| rs3887925 | T | C | 0.059 | 2.90E-23 |
| rs1260326 | C | T | 0.064 | 1.60E-22 |
| rs3806155 | T | A | 0.176 | 3.30E-22 |
| rs1800961 | T | C | 0.166 | 2.70E-21 |
| rs10770142 | G | C | 0.063 | 4.00E-21 |
| rs8192675 | T | C | 0.066 | 5.80E-21 |
| rs12578595 | C | T | 0.074 | 3.10E-20 |
| rs243018 | G | C | 0.059 | 1.80E-19 |
| rs10184004 | C | T | 0.059 | 2.00E-19 |
| rs6496609 | C | A | 0.064 | 2.80E-19 |
| rs34715063 | C | T | 0.088 | 5.00E-19 |
| rs340874 | C | T | 0.055 | 1.10E-18 |
| rs2366214 | A | G | 0.059 | 1.70E-18 |
| rs7732130 | G | A | 0.061 | 5.70E-18 |
| rs2859885 | C | T | 0.068 | 1.60E-17 |
| rs2820446 | C | G | 0.059 | 2.80E-17 |
| rs234866 | G | A | 0.049 | 3.70E-17 |
| rs11759026 | G | A | 0.063 | 4.50E-17 |
| rs7100404 | C | T | 0.046 | 4.70E-17 |
| rs6831006 | G | C | 0.055 | 8.50E-17 |
| rs343093 | G | C | 0.075 | 1.60E-16 |
| rs17168486 | T | C | 0.068 | 3.30E-16 |
| rs11688682 | G | C | 0.061 | 5.80E-16 |
| rs6813195 | C | T | 0.058 | 7.00E-16 |
| rs1562396 | G | A | 0.056 | 9.60E-16 |
| rs6567160 | C | T | 0.061 | 1.00E-15 |
| rs35895680 | C | A | 0.055 | 1.10E-15 |
| rs13094957 | T | C | 0.063 | 1.60E-15 |
| rs878521 | A | G | 0.059 | 2.30E-15 |
| rs429358 | T | C | 0.072 | 2.50E-15 |
| rs12625671 | C | T | 0.079 | 3.30E-15 |
| rs10406431 | A | G | 0.05 | 3.80E-15 |
| rs835576 | C | T | 0.079 | 5.80E-15 |
| rs7081841 | G | C | 0.053 | 6.00E-15 |
| rs2925979 | T | C | 0.055 | 7.10E-15 |
| rs2307111 | T | C | 0.05 | 1.00E-14 |
| rs13257021 | A | G | 0.049 | 3.10E-14 |
| rs1127215 | C | T | 0.049 | 3.90E-14 |
| rs7970350 | T | C | 0.048 | 3.90E-14 |
| rs3798519 | C | A | 0.062 | 4.60E-14 |
| rs2421016 | C | T | 0.048 | 5.00E-14 |
| rs2642588 | G | T | 0.053 | 5.20E-14 |
| rs1996546 | G | T | 0.069 | 8.90E-14 |
| rs12789028 | A | G | 0.061 | 1.10E-13 |
| rs6458354 | C | T | 0.052 | 1.40E-13 |
| rs348330 | G | A | 0.049 | 2.10E-13 |
| rs13290396 | C | T | 0.094 | 2.30E-13 |
| rs3890400 | A | G | 0.05 | 3.00E-13 |
| rs508419 | G | A | 0.05 | 3.10E-13 |
| rs8008910 | A | G | 0.055 | 4.20E-13 |
| rs58542926 | T | C | 0.087 | 4.70E-13 |
| rs2292662 | C | T | 0.064 | 4.80E-13 |
| rs2890156 | A | T | 0.066 | 6.00E-13 |
| rs7163757 | C | T | 0.047 | 7.90E-13 |
| rs66815886 | G | T | 0.051 | 8.70E-13 |
| rs7313668 | T | G | 0.046 | 9.90E-13 |
| rs6485462 | C | T | 0.049 | 1.60E-12 |
| rs1426371 | G | A | 0.052 | 1.70E-12 |
| rs2115107 | A | G | 0.047 | 1.70E-12 |
| rs12454712 | T | C | 0.048 | 2.40E-12 |
| rs6752053 | T | C | 0.045 | 3.20E-12 |
| rs36575 | C | T | 0.08 | 3.30E-12 |
| rs6937795 | A | C | 0.045 | 3.70E-12 |
| rs505922 | C | T | 0.047 | 3.70E-12 |
| rs6600191 | T | C | 0.059 | 4.50E-12 |
| rs8071043 | C | T | 0.047 | 7.50E-12 |
| rs2283164 | A | G | 0.102 | 9.10E-12 |
| rs2800733 | A | G | 0.049 | 9.60E-12 |
| rs1473781 | A | G | 0.046 | 1.00E-11 |
| rs11819995 | T | C | 0.053 | 1.10E-11 |
| rs12811407 | A | G | 0.048 | 1.20E-11 |
| rs684214 | T | C | 0.048 | 1.20E-11 |
| rs539515 | C | A | 0.055 | 1.40E-11 |
| rs41277236 | T | C | 0.112 | 2.00E-11 |
| rs11020308 | A | C | 0.044 | 2.10E-11 |
| rs1426385 | A | G | 0.043 | 2.30E-11 |
| rs4240673 | T | C | 0.043 | 2.30E-11 |
| rs862320 | C | T | 0.043 | 2.60E-11 |
| rs7819706 | A | G | 0.067 | 2.80E-11 |
| rs145003494 | A | G | 0.227 | 3.10E-11 |
| rs3111316 | A | G | 0.044 | 3.50E-11 |
| rs10848960 | G | C | 0.051 | 4.20E-11 |
| rs62107261 | T | C | 0.105 | 6.50E-11 |
| rs78470967 | T | A | 0.113 | 6.60E-11 |
| rs1108646 | A | G | 0.044 | 6.80E-11 |
| rs879882 | C | T | 0.042 | 7.20E-11 |
| rs9957320 | G | T | 0.055 | 7.50E-11 |
| rs76547628 | T | C | 0.054 | 8.40E-11 |
| rs13130484 | T | C | 0.044 | 8.50E-11 |
| rs3918298 | G | A | 0.135 | 1.20E-10 |
| rs145678014 | G | T | 0.105 | 1.40E-10 |
| rs736266 | T | A | 0.041 | 1.40E-10 |
| rs3094515 | C | T | 0.048 | 1.60E-10 |
| rs62492368 | A | G | 0.044 | 1.90E-10 |
| rs4911405 | T | C | 0.043 | 2.90E-10 |
| rs11642430 | G | C | 0.041 | 3.30E-10 |
| rs3810291 | A | G | 0.043 | 3.40E-10 |
| rs73221123 | T | C | 0.098 | 4.00E-10 |
| rs9899520 | A | G | 0.05 | 4.20E-10 |
| rs539298 | A | G | 0.04 | 4.60E-10 |
| rs2858980 | G | A | 0.054 | 4.60E-10 |
| rs4237150 | C | G | 0.04 | 4.80E-10 |
| rs6821438 | A | G | 0.04 | 5.10E-10 |
| rs34584161 | A | G | 0.047 | 5.40E-10 |
| rs10842708 | G | A | 0.045 | 6.40E-10 |
| rs6063046 | A | G | 0.045 | 7.30E-10 |
| rs6884702 | G | A | 0.04 | 7.90E-10 |
| rs10188334 | C | T | 0.052 | 8.10E-10 |
| rs10893827 | A | G | 0.056 | 9.00E-10 |
| rs76435632 | G | C | 0.108 | 9.80E-10 |
| rs917195 | C | T | 0.047 | 1.00E-09 |
| rs12001437 | C | T | 0.04 | 1.10E-09 |
| rs114322470 | T | G | 0.149 | 1.10E-09 |
| rs1020731 | A | G | 0.043 | 1.30E-09 |
| rs7178762 | C | T | 0.039 | 1.40E-09 |
| rs7781557 | C | T | 0.05 | 1.60E-09 |
| rs11636031 | T | C | 0.046 | 1.60E-09 |
| rs30614 | A | G | 0.039 | 1.70E-09 |
| rs12380322 | G | A | 0.039 | 1.90E-09 |
| rs57676627 | T | C | 0.057 | 2.00E-09 |
| rs10406327 | C | G | 0.038 | 2.40E-09 |
| rs28691713 | C | T | 0.041 | 2.40E-09 |
| rs12345069 | C | T | 0.043 | 3.40E-09 |
| rs12073283 | C | G | 0.066 | 3.60E-09 |
| rs4709746 | C | T | 0.057 | 4.00E-09 |
| rs2066827 | G | T | 0.047 | 4.40E-09 |
| rs73347525 | A | G | 0.05 | 4.90E-09 |
| rs1665901 | A | T | 0.04 | 5.00E-09 |
| rs6905288 | A | G | 0.038 | 5.80E-09 |
| rs9873519 | T | C | 0.037 | 6.30E-09 |
| rs10471048 | G | C | 0.039 | 6.40E-09 |
| rs34965774 | A | G | 0.054 | 6.40E-09 |
| rs262549 | G | C | 0.047 | 6.50E-09 |
| rs12680692 | A | T | 0.041 | 6.60E-09 |
| rs1412234 | C | T | 0.039 | 7.70E-09 |
| rs76263492 | T | G | 0.09 | 8.90E-09 |
| rs4733612 | G | A | 0.042 | 9.20E-09 |
| rs12920022 | A | T | 0.053 | 1.00E-08 |
| rs7594480 | T | C | 0.075 | 1.10E-08 |
| rs10935897 | A | G | 0.036 | 1.20E-08 |
| rs738408 | T | C | 0.044 | 1.20E-08 |
| rs7104712 | C | A | 0.04 | 1.50E-08 |
| rs34298980 | T | C | 0.038 | 1.60E-08 |
| rs329122 | A | G | 0.037 | 1.70E-08 |
| rs4776970 | A | T | 0.038 | 1.70E-08 |
| rs4258054 | T | C | 0.04 | 2.60E-08 |
| rs55928417 | G | T | 0.036 | 2.70E-08 |
| rs17261179 | T | C | 0.036 | 2.70E-08 |
| rs1790116 | T | G | 0.043 | 3.10E-08 |
| rs2624847 | G | T | 0.041 | 3.50E-08 |
| rs963740 | A | T | 0.039 | 3.70E-08 |
| rs12419690 | G | A | 0.035 | 3.90E-08 |
| rs223423 | G | A | 0.035 | 4.30E-08 |
| rs10882099 | T | C | 0.019 | 4.50E-08 |
| rs12883788 | T | C | 0.035 | 4.60E-08 |
| rs7903146 | T | C | 0.298 | 1.00E-200 |

# Table S4. Multiple imputation for missing data.

| Variables | Complete | Incomplete | Imputed | Total |
| --- | --- | --- | --- | --- |
| Age, years | 145,998 | 0 | 0 | 145,998 |
| Sex, n (%) | 145,998 | 0 | 0 | 145,998 |
| Townsend Deprivation Index | 145,792 | 206 | 206 | 145,998 |
| Smoking status, % | 145,544 | 454 | 454 | 145,998 |
| Alcohol Consumption Status, % | 145,878 | 120 | 120 | 145,998 |
| Employment | 145,635 | 363 | 363 | 145,998 |
| Raw grip strength | 145,681 | 317 | 317 | 145,998 |
| Fat free mass | 143,669 | 2,329 | 2,329 | 145,998 |
| Beef intake, times/week (average) | 145,544 | 454 | 454 | 145,998 |
| Lamb intake, times/week (average) | 145,220 | 778 | 778 | 145,998 |
| Pork intake, times/week (average) | 145,260 | 738 | 738 | 145,998 |
| Oil Fish intake, times/week | 145,337 | 661 | 661 | 145,998 |
| Non-oil Fish intake, times/week | 145,519 | 479 | 479 | 145,998 |
| Physical activity (MET-minutes/week) | 145,998 | 0 | 0 | 145,998 |
| Polygenic risk scores for type 2 diabetes | 145,998 | 0 | 0 | 145,998 |

# Table S5. Associations of muscle strength and incident type 2 diabetes stratified by different levels of genetic susceptibility to type 2 diabetes after excluding type 2 diabetes accrued in the first 4 years of follow up.

| Categories  of T2D genetic risk | Categories  of muscle strength | Number of Participants | Number of T2D events | Crude incident rate per 100,000-person years | HR (95% CI) | P for interaction^1^ |
| --- | --- | --- | --- | --- | --- | --- |
| Low | Low (Reference) | 9,278 | 186 | 267.6 | 1 (Reference) | Additive: 0.003  Multiplicative: 0.618 |
|  | Medium | 9,452 | 132 | 184.0 | 0.72 (0.57, 0.90) |  |
|  | High | 9,440 | 94 | 128.2 | 0.53 (0.41, 0.68) |  |
| Medium | Low (Reference) | 27,983 | 838 | 401.4 | 1 (Reference) |  |
|  | Medium | 28,147 | 576 | 270.4 | 0.72 (0.65, 0.80) |  |
|  | High | 28,146 | 489 | 223.3 | 0.62 (0.55, 0.69) |  |
| High | Low (Reference) | 9,253 | 388 | 563.9 | 1 (Reference) |  |
|  | Medium | 9,218 | 310 | 444.7 | 0.82 (0.71, 0.95) |  |
|  | High | 9,428 | 227 | 310.6 | 0.58 (0.49, 0.68) |  |

Note: Cox regression models with age as the underlying timescale were adjusted for sex, Townsend Deprivation Index, employment status, tobacco smoking status, alcohol drinking status, red meat intake, fish intake, physical activity, genotype array type and the first twenty principal components. Abbreviations: T2D, type 2 diabetes; HR: hazard ratio; CI: confidence interval.

^1^P for interaction: the p-value for interaction term between categories of muscle strength and genetic risk for T2D (both treated as ordinal variables) for incident T2D on additive and multiplicative scales.

# Table S6. Associations of muscle strength and incident type 2 diabetes stratified by different levels of genetic susceptibility to type 2 diabetes after excluding individuals with the 2^nd^-degree (or higher) genetic relatedness.

| Categories  of T2D genetic risk | Categories  of muscle strength | Number of Participants | Number of T2D events | Crude incident rate per 100,000-person years | HR (95% CI) | P for interaction^1^ |
| --- | --- | --- | --- | --- | --- | --- |
| Low | Low (Reference) | 8,957 | 277 | 415.7 | 1 (Reference) | Additive: 0.008  Multiplicative: 0.064 |
|  | Medium | 9,085 | 182 | 264.9 | 0.68 (0.57, 0.82) |  |
|  | High | 9,042 | 124 | 177.0 | 0.46 (0.37, 0.57) |  |
| Medium | Low (Reference) | 27,016 | 1,179 | 590.2 | 1 (Reference) |  |
|  | Medium | 27,095 | 811 | 397.8 | 0.72 (0.66, 0.79) |  |
|  | High | 27,046 | 622 | 296.6 | 0.56 (0.51, 0.62) |  |
| High | Low (Reference) | 9,061 | 566 | 851.1 | 1 (Reference) |  |
|  | Medium | 8,936 | 444 | 664.0 | 0.79 (0.70, 0.90) |  |
|  | High | 9,130 | 331 | 471.0 | 0.58 (0.50, 0.66) |  |

Note: Cox regression models with age as the underlying timescale were adjusted for sex, Townsend Deprivation Index, employment status, tobacco smoking status, alcohol drinking status, red meat intake, fish intake, physical activity, genotype array type and the first twenty principal components. Abbreviations: T2D, type 2 diabetes; HR: hazard ratio; CI: confidence interval.

^1^P for interaction: the p-value for the interaction term between categories of muscle strength and genetic risk for T2D (both treated as ordinal variables) for incident T2D on additive and multiplicative scales.

# Table S7. Associations of absolute muscle strength (measured grip strength) and incident type 2 diabetes stratified by different levels of genetic susceptibility to type 2 diabetes.

| Categories  of T2D genetic risk | Categories  of muscle strength | Number of Participants | Number of T2D events | Crude incident rate per 100,000-person years | HR (95% CI) | P for interaction^1^ |
| --- | --- | --- | --- | --- | --- | --- |
| Low | Low (Reference) | 9,095 | 225 | 331.1 | 1 (Reference) | Additive: 0.081  Multiplicative: 0.683 |
|  | Medium | 9,190 | 193 | 277.4 | 0.89 (0.73, 1.08) |  |
|  | High | 10,082 | 191 | 246.1 | 0.82 (0.67, 1.01) |  |
| Medium | Low (Reference) | 27,626 | 1,015 | 494.3 | 1 (Reference) |  |
|  | Medium | 27,424 | 832 | 402.9 | 0.93 (0.85, 1.02) |  |
|  | High | 30,056 | 886 | 382.8 | 0.90 (0.82, 0.99) |  |
| High | Low (Reference) | 9,183 | 511 | 753.6 | 1 (Reference) |  |
|  | Medium | 9,061 | 424 | 639.7 | 0.93 (0.83, 1.07) |  |
|  | High | 10,131 | 456 | 589.2 | 0.87 (0.77, 0.99) |  |

Note: Cox regression models with age as the underlying timescale were adjusted for sex, Townsend Deprivation Index, employment status, tobacco smoking status, alcohol drinking status, red meat intake, fish intake, BMI, physical activity, genotype array type and the first twenty principal components. Abbreviations: T2D, type 2 diabetes; HR: hazard ratio; CI: confidence interval.

^1^P for interaction: the p-value for the interaction term between categories of muscle strength and genetic risk for T2D (both treated as ordinal variables) for incident T2D on additive and multiplicative scales.

# Table S8. Associations of muscle strength and incident type 2 diabetes stratified by different levels of genetic susceptibility to type 2 diabetes using a less stringent linkage disequilibrium cut-off (r^2^<0.01) combined with the same genome-wide significance level (i.e., *P* <5×10^-8^) in the clumping procedure.

| Categories  of T2D genetic risk | Categories  of muscle strength | Number of Participants | Number of T2D events | Crude incident rate per 100,000-person years | HR (95% CI) | P for interaction^1^ |
| --- | --- | --- | --- | --- | --- | --- |
| Low | Low (Reference) | 9,484 | 265 | 375.6 | 1 (Reference) | Additive:<0.001  Multiplicative: 0.121 |
|  | Medium | 9,446 | 185 | 258.8 | 0.73 (0.61, 0.88) |  |
|  | High | 9,437 | 130 | 178.0 | 0.53 (0.43, 0.65) |  |
| Medium | Low (Reference) | 28,316 | 1,246 | 595.0 | 1 (Reference) |  |
|  | Medium | 28,476 | 839 | 391.4 | 0.70 (0.64, 0.77) |  |
|  | High | 28,319 | 639 | 290.9 | 0.54 (0.49, 0.60) |  |
| High | Low (Reference) | 9,421 | 608 | 878.2 | 1 (Reference) |  |
|  | Medium | 9,364 | 463 | 661.2 | 0.78 (0.69, 0.88) |  |
|  | High | 9,585 | 368 | 499.5 | 0.60 (0.53, 0.68) |  |

Note: Cox regression models with age as the underlying timescale were adjusted for sex, Townsend Deprivation Index, employment status, tobacco smoking status, alcohol drinking status, red meat intake, fish intake, physical activity, genotype array type and the first twenty principal components. Abbreviations: T2D, type 2 diabetes; HR: hazard ratio; CI: confidence interval.

^1^P for interaction: the p-value for the interaction term between categories of muscle strength and genetic risk for T2D (both treated as ordinal variables) for incident T2D on additive and multiplicative scales.

# Table S9. Associations of muscle strength and incident type 2 diabetes stratified by different levels of genetic susceptibility to type 2 diabetes using incident T2D ascertained only based on primary care data and using the mid-point between the last primary care consultation date without T2D and the date of the first T2D as the incident date of T2D.

| Categories  of T2D genetic risk | Categories  of muscle strength | Number of Participants | Number of T2D events | Crude incident rate per 100,000-person years | HR (95% CI) | P for interaction^1^ |
| --- | --- | --- | --- | --- | --- | --- |
| Low | Low (Reference) | 9,380 | 255 | 364.8 | 1 (Reference) | Additive: 0.016  Multiplicative: 0.030 |
|  | Medium | 9,513 | 173 | 240.4 | 0.70 (0.57, 0.85) |  |
|  | High | 9,474 | 115 | 156.6 | 0.48 (0.38, 0.60) |  |
| Medium | Low (Reference) | 28,406 | 1,111 | 528.4 | 1 (Reference) |  |
|  | Medium | 28,315 | 749 | 350.0 | 0.71 (0.64, 0.78) |  |
|  | High | 28,385 | 596 | 271.3 | 0.57 (0.51, 0.63) |  |
| High | Low (Reference) | 9,456 | 542 | 779.0 | 1 (Reference) |  |
|  | Medium | 9,552 | 424 | 603.7 | 0.81 (0.71, 0.92) |  |
|  | High | 9,456 | 335 | 455.8 | 0.62 (0.54, 0.71) |  |

Note: Cox regression models with age as the underlying timescale were adjusted for sex, Townsend Deprivation Index, employment status, tobacco smoking status, alcohol drinking status, red meat intake, fish intake, physical activity, genotype array type and the first twenty principal components. Abbreviations: T2D, type 2 diabetes; HR: hazard ratio; CI: confidence interval.

^1^P for interaction: the p-value for the interaction term between categories of muscle strength and genetic risk for T2D (both treated as ordinal variables) for incident T2D on additive and multiplicative scales.

# Table S10. Associations of muscle strength and incident type 2 diabetes stratified by different levels of genetic susceptibility to type 2 diabetes using incident T2D ascertained only based on hospital admission records and UK death registries data.

| Categories  of T2D genetic risk | Categories  of muscle strength | Number of Participants | Number of T2D events | Crude incident rate per 100,000-person years | HR (95% CI) | P for interaction^1^ |
| --- | --- | --- | --- | --- | --- | --- |
| Low | Low (Reference) | 20,644 | 770 | 273.7 | 1 (Reference) | Additive: <0.001  Multiplicative: 0.003 |
|  | Medium | 20,820 | 425 | 148.1 | 0.59 (0.53, 0.69) |  |
|  | High | 20,899 | 260 | 89.1 | 0.37 (0.32, 0.43) |  |
| Medium | Low (Reference) | 62,372 | 3,378 | 400.4 | 1 (Reference) |  |
|  | Medium | 62,624 | 2,069 | 240.8 | 0.65 (0.61, 0.68) |  |
|  | High | 62,215 | 1,361 | 157.1 | 0.44 (0.41, 0.47) |  |
| High | Low (Reference) | 20,656 | 1,650 | 593.4 | 1 (Reference) |  |
|  | Medium | 20,990 | 1,106 | 393.3 | 0.70 (0.65, 0.76) |  |
|  | High | 20,752 | 723 | 248.6 | 0.46 (0.42, 0.50) |  |

Note: Cox regression models with age as the underlying timescale were adjusted for sex, Townsend Deprivation Index, employment status, tobacco smoking status, alcohol drinking status, red meat intake, fish intake, physical activity, genotype array type and the first twenty principal components. Abbreviations: T2D, type 2 diabetes; HR: hazard ratio; CI: confidence interval.

^1^P for interaction: the p-value for the interaction term between categories of muscle strength and genetic risk for T2D (both treated as ordinal variables) for incident T2D on additive and multiplicative scales.

# Table S11. Associations of muscle strength and incident type 2 diabetes stratified by different levels of genetic susceptibility to type 2 diabetes using values imputed for the covariates missing, assuming data missing at random.

| Categories  of T2D genetic risk | Categories  of muscle strength | Number of Participants | Number of T2D events | Crude incident rate per 100,000-person years | HR (95% CI) | P for interaction^1^ |
| --- | --- | --- | --- | --- | --- | --- |
| Low | Low (Reference) | 9,659 | 299 | 415.9 | 1 (Reference) | Additive: 0.010  Multiplicative: 0.043 |
|  | Medium | 9,797 | 201 | 271.6 | 0.71 (0.58, 0.85) |  |
|  | High | 9,741 | 136 | 180.3 | 0.49 (0.40, 0.61) |  |
| Medium | Low (Reference) | 29,220 | 1,288 | 596.1 | 1 (Reference) |  |
|  | Medium | 29,200 | 870 | 396.1 | 0.72 (0.65, 0.78) |  |
|  | High | 29,176 | 689 | 304.8 | 0.57 (0.52, 0.63) |  |
| High | Low (Reference) | 9,764 | 618 | 862.0 | 1 (Reference) |  |
|  | Medium | 9,601 | 474 | 659.1 | 0.80 (0.71, 0.91) |  |
|  | High | 9,834 | 364 | 481.6 | 0.62 (0.54, 0.71) |  |

Note: Cox regression models with age as the underlying timescale were adjusted for sex, Townsend Deprivation Index, employment status, tobacco smoking status, alcohol drinking status, red meat intake, fish intake, physical activity, genotype array type and the first twenty principal components. Abbreviations: T2D, type 2 diabetes; HR: hazard ratio; CI: confidence interval.

^1^P for interaction: the p-value for the interaction term between categories of muscle strength and genetic risk for T2D (both treated as ordinal variables) for incident T2D on additive and multiplicative scales.

# Table S12. Associations of muscle strength quintiles and genetic susceptibility to type 2 diabetes with incident type 2 diabetes.

| Muscle strength quintiles | Number of participants | Number of cases | Crude incident rate per 100,000-person years | Hazard ratio  (95% confidence interval) |
| --- | --- | --- | --- | --- |
|  |  |  |  |  |
| Lowest 20% (Reference) | 28,340 | 1,454 | 697.3 | 1 (Reference) |
| 20%-40% | 28,386 | 980 | 462.8 | 0.70 (0.65, 0.76) |
| 40%-60% | 28,346 | 911 | 427.1 | 0.66 (0.61, 0.72) |
| 60%-80% | 28,395 | 768 | 353.8 | 0.56 (0.51, 0.61) |
| Top 20% | 28,381 | 630 | 284.5 | 0.46 (0.42, 0.50) |

Note: Cox regression models with age as the underlying timescale were adjusted for sex, Townsend Deprivation Index, employment status, tobacco smoking status, alcohol drinking status, red meat intake, fish intake, physical activity, polygenic risk score for T2D, genotype array type, and the first twenty principal components. Abbreviations: T2D, type 2 diabetes.

# Figure S1. Participants’ flow chart.

Note: participants were asked to indicate their ethnic background by choosing from the following categories (UK Biobank Data-Field: 21000): ‘White’, ‘Mixed’, ‘Asian or Asian British’, ‘Black or Black British’, ‘Chinese’, ‘Other ethnic group’, ‘Do not know’, and ‘Prefer not to answer’.


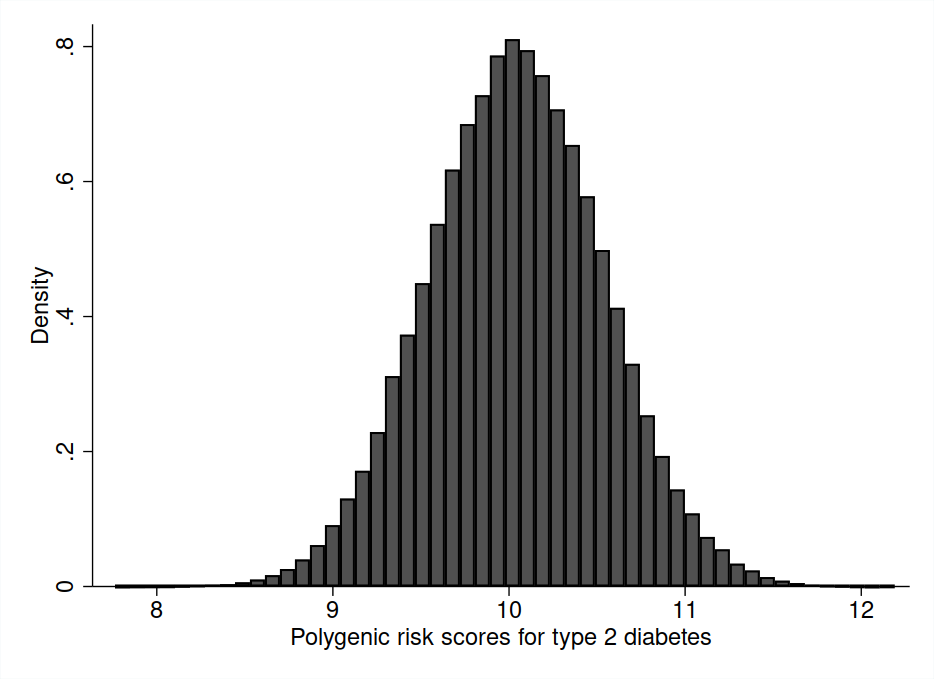


# Figure S2. Distribution of polygenic risk scores (PRSs) for type 2 diabetes using 138 uncorrelated SNPs (genome-wide significant at *P* <5×10^-8^ and in low linkage disequilibrium defined according to *r^2^*<0.001).


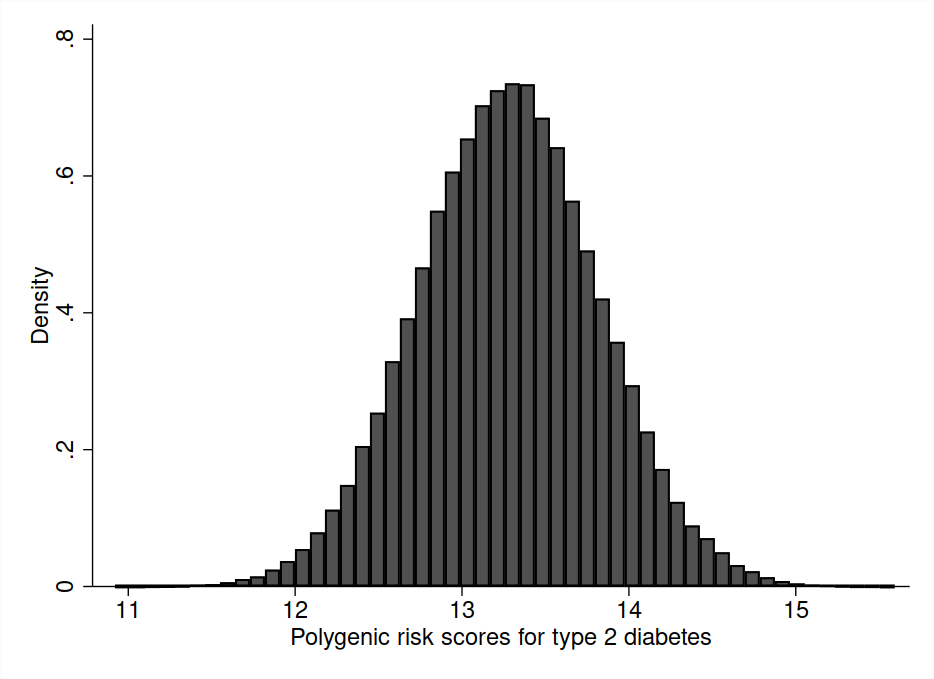


# Figure S3. Distribution of polygenic risk scores (PRSs) for type 2 diabetes using 187 uncorrelated SNPs (genome-wide significant at *P* <5×10^-8^ and in low linkage disequilibrium defined according to *r^2^*<0.01).
